# Supplementary figures and images for: Centriolar satellites expedite mother centriole remodeling to promote ciliogenesis
Source: eLife. 2023 Feb 15;12:e79299. doi: 10.7554/eLife.79299 (PMC9998092; doi:10.7554/eLife.79299)

Figure 1 A

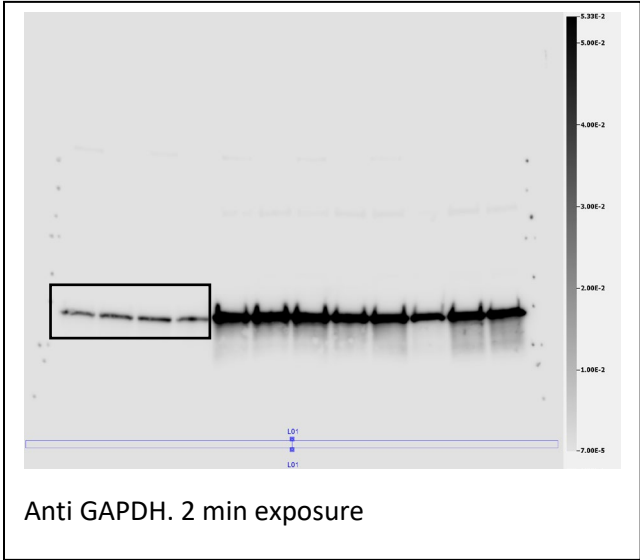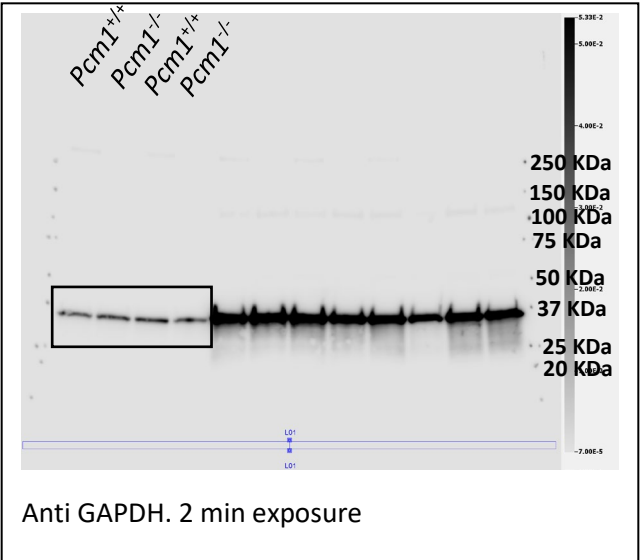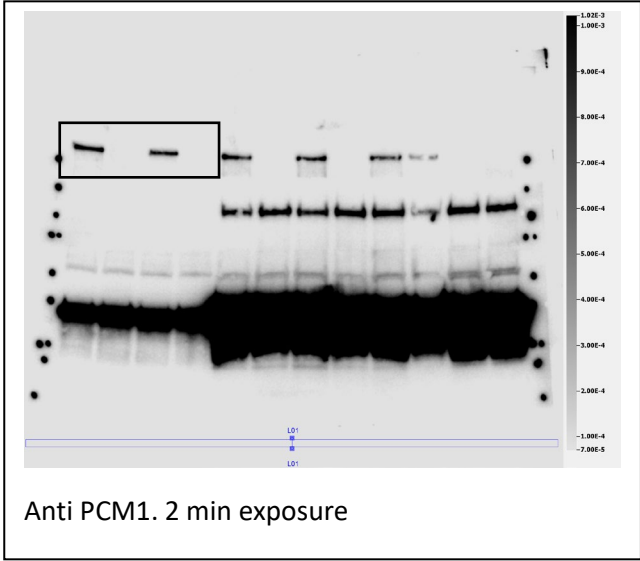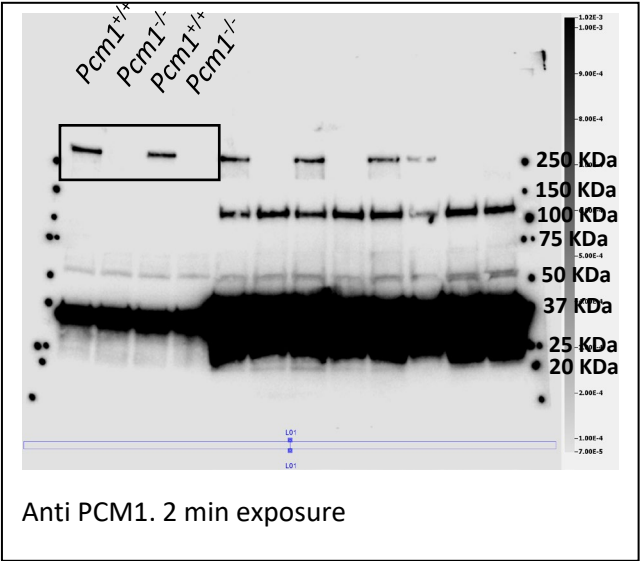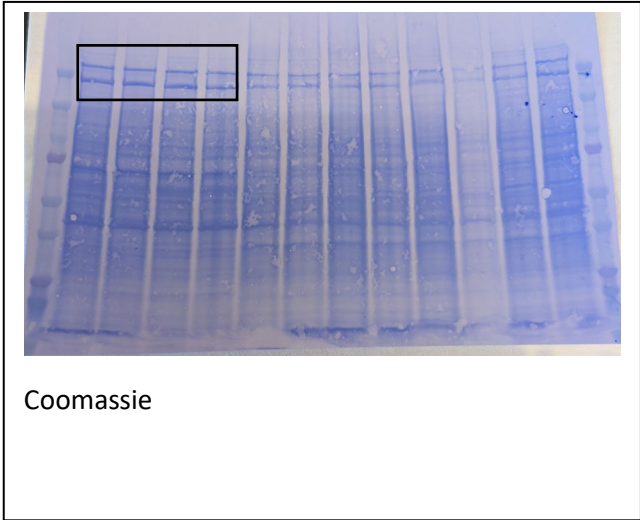

Figure 1 Figure Supplement 1B

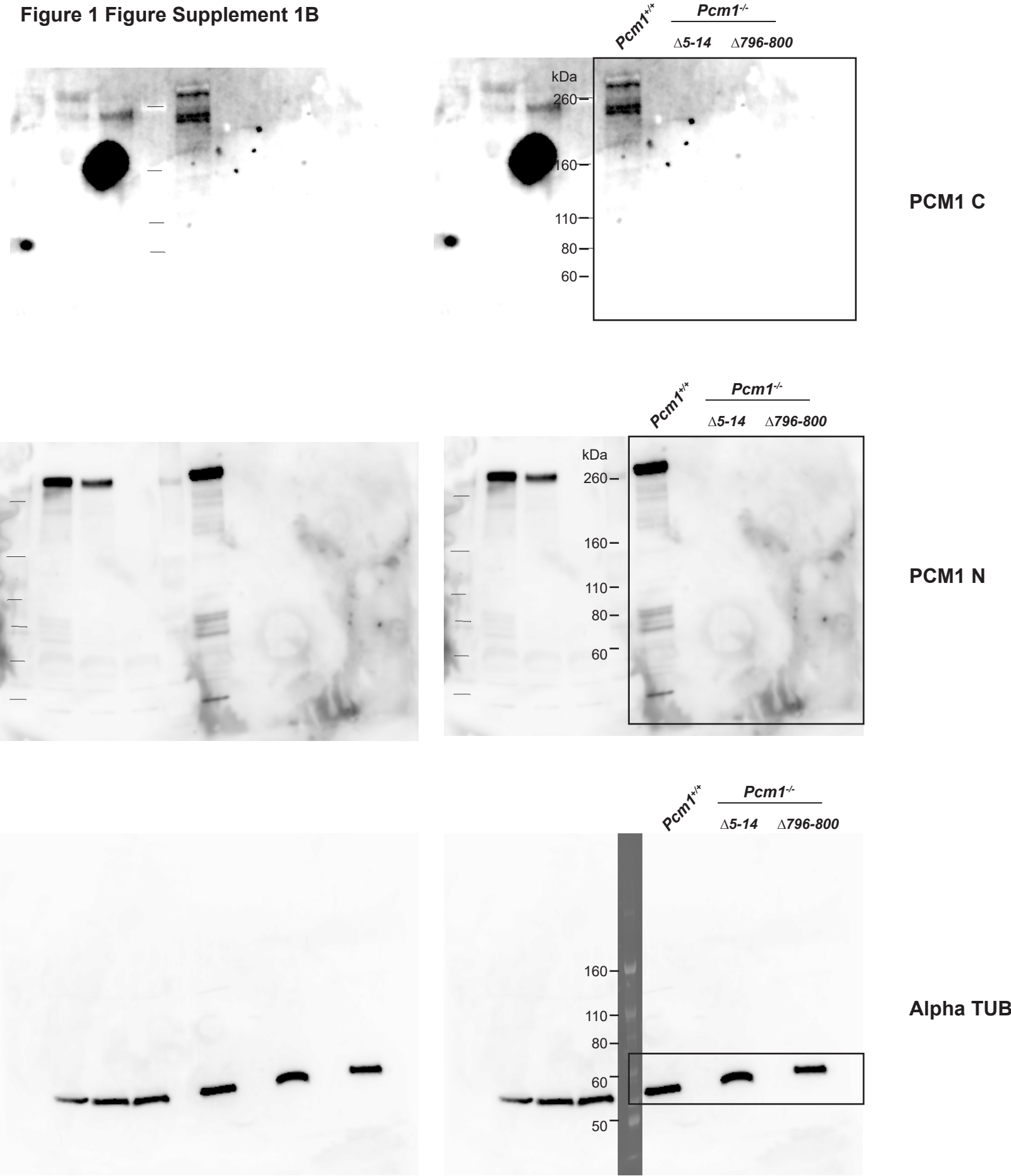

Supplement: Figure 1—source data 1. [file elife-79299-fig1-data1.pdf]

Figure 4 G

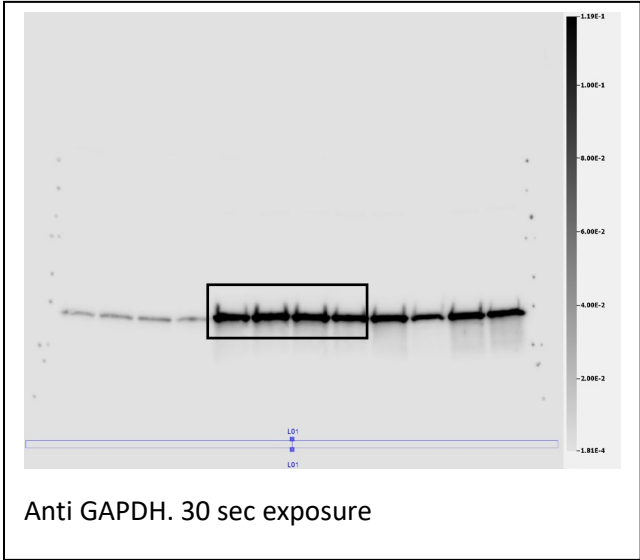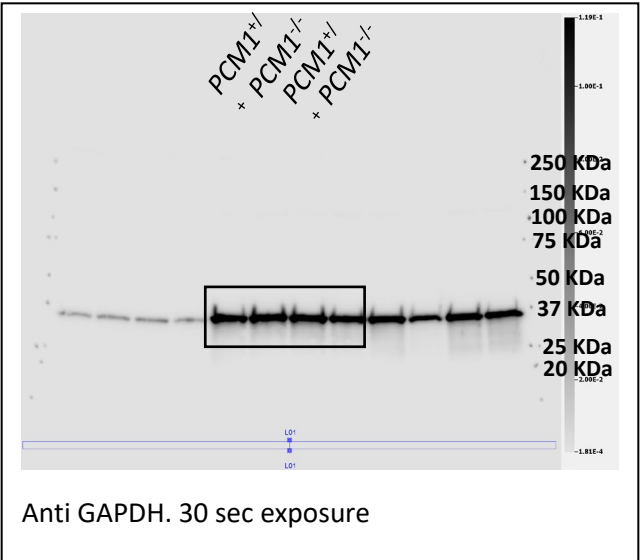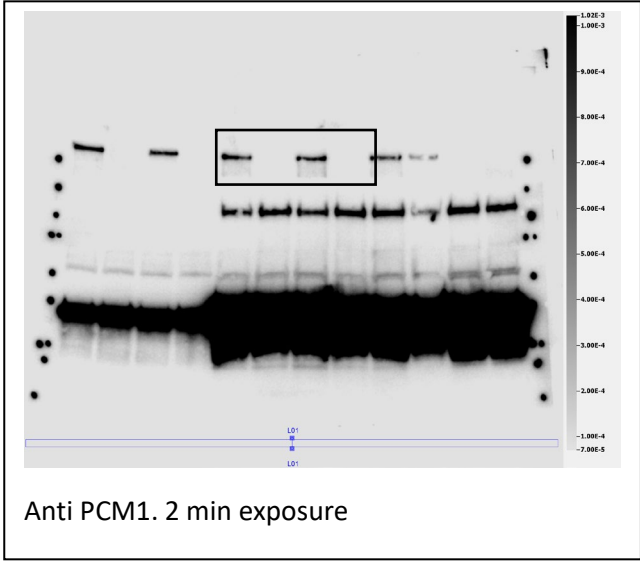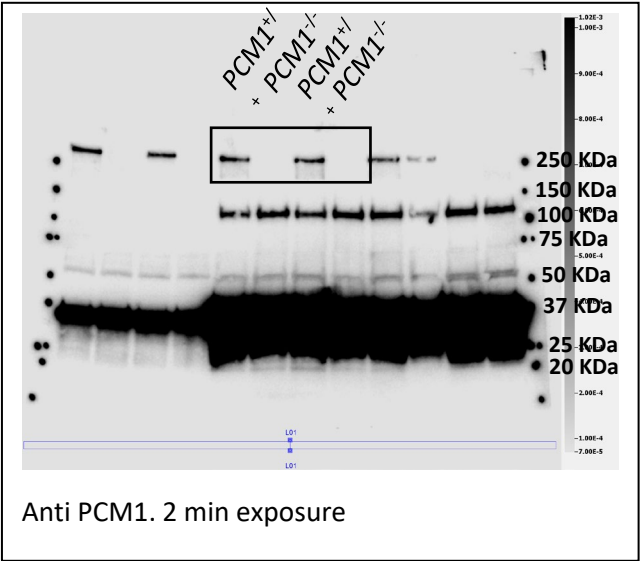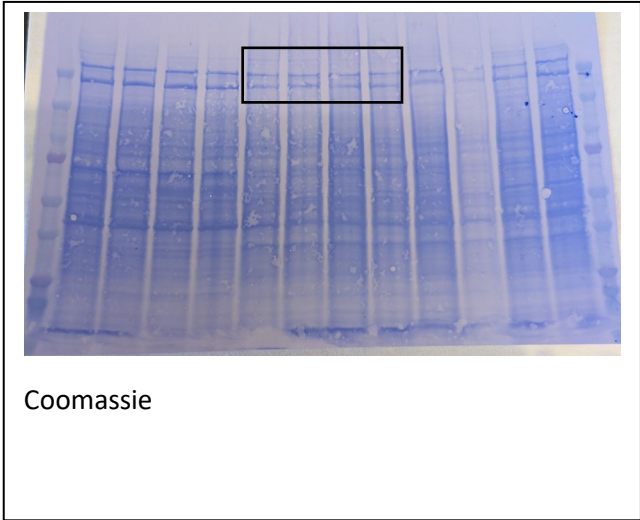

Supplement: Figure 4—source data 1. [file elife-79299-fig4-data1.pdf]

Figure 8 C

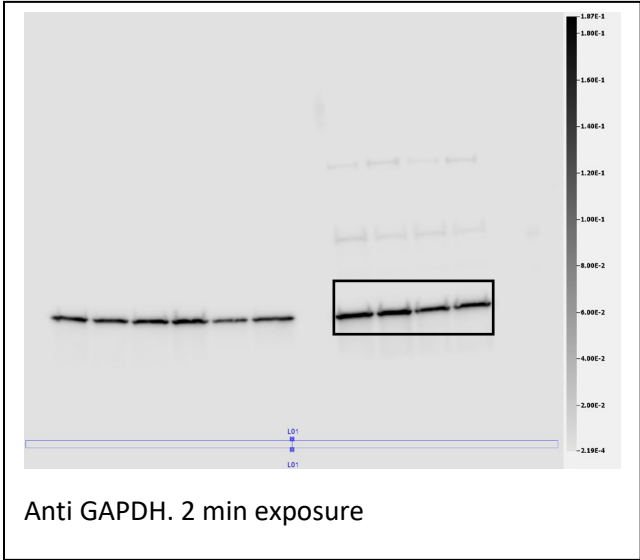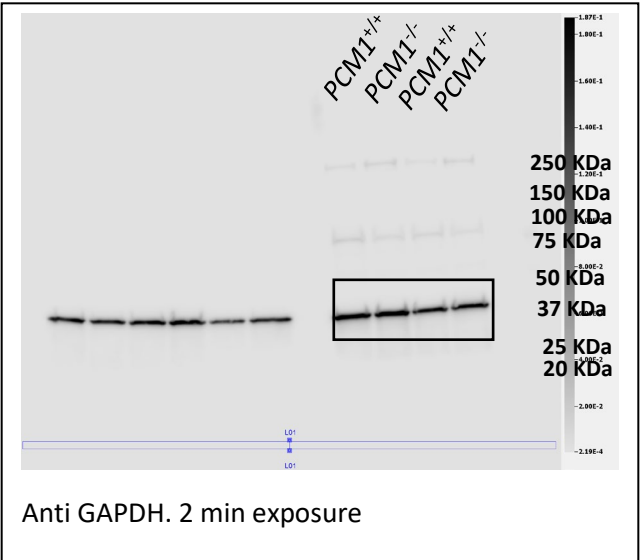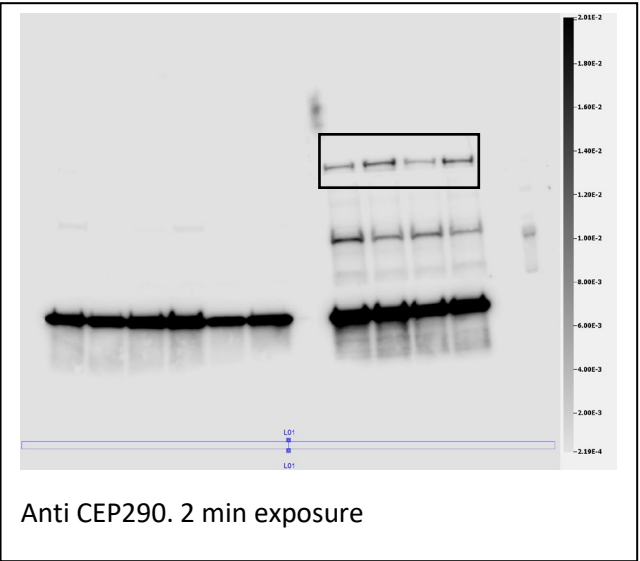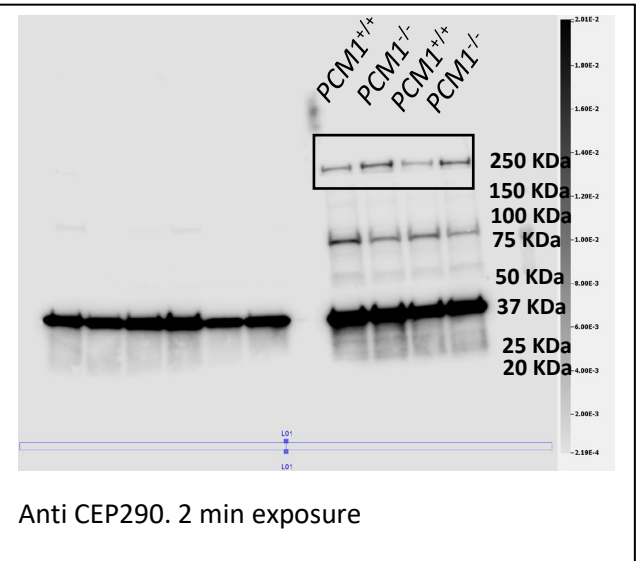

Figure 8 C

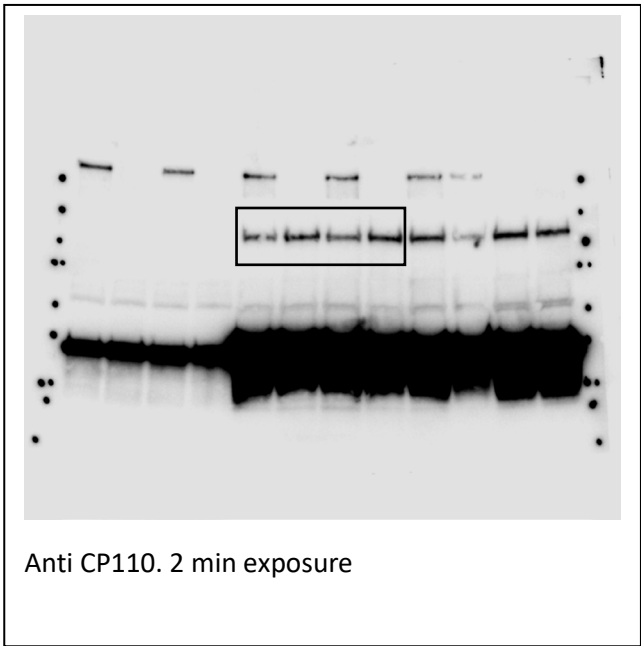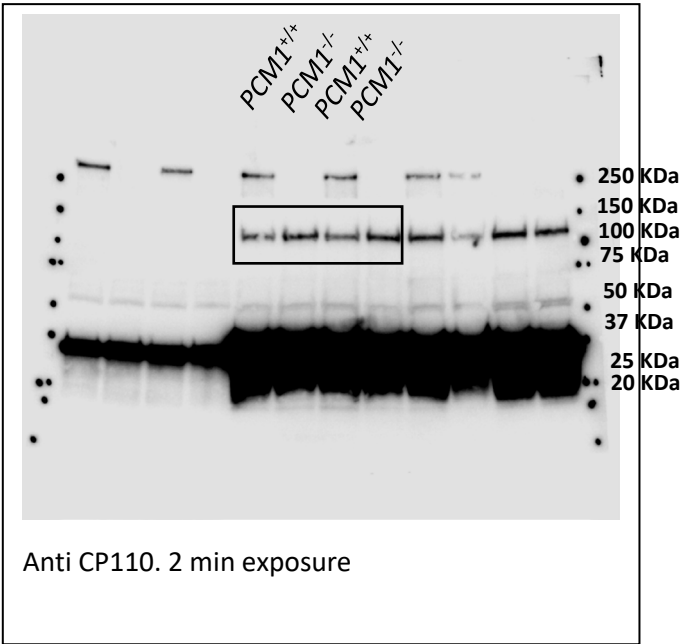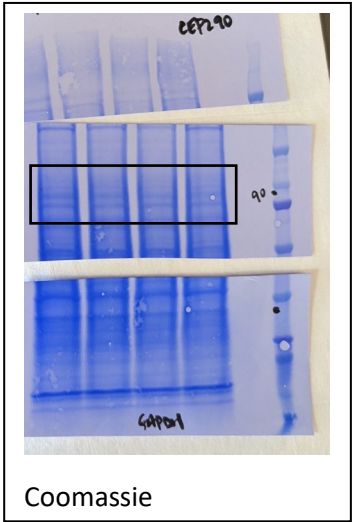

Figure 8 I

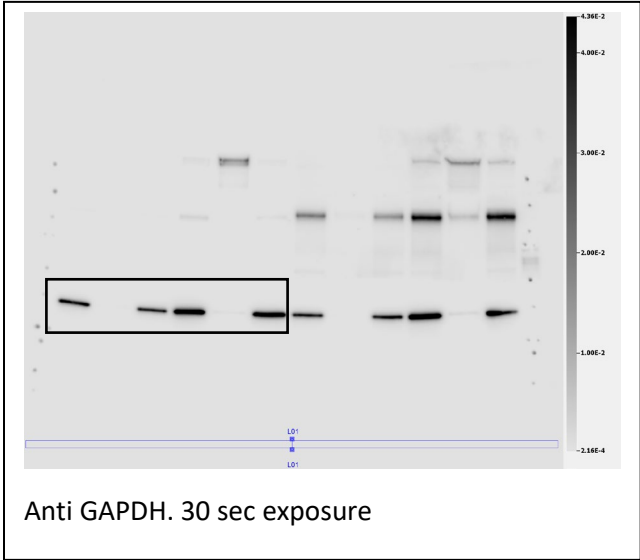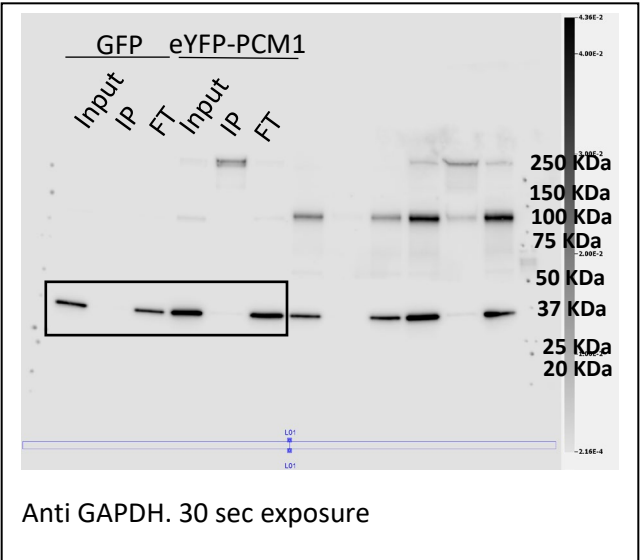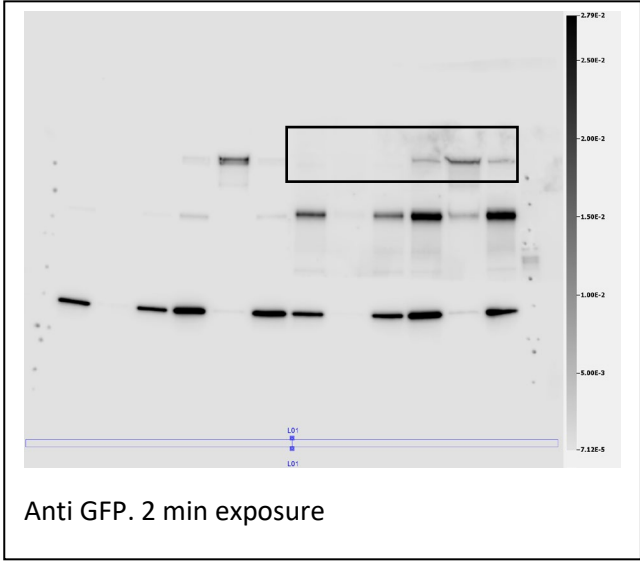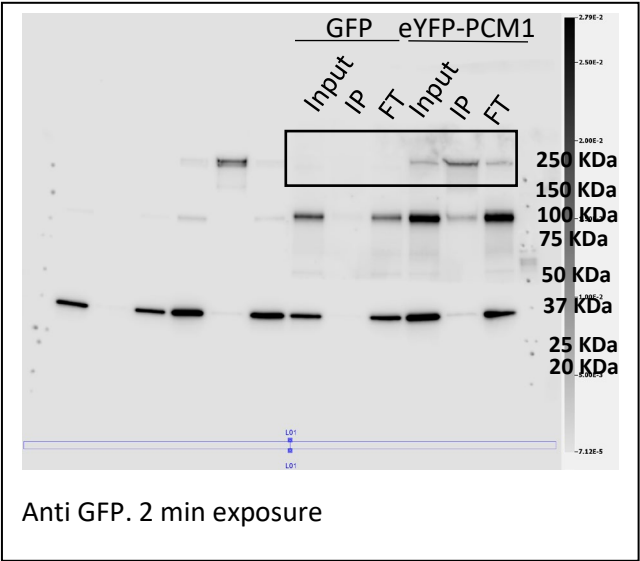

Figure 8 I

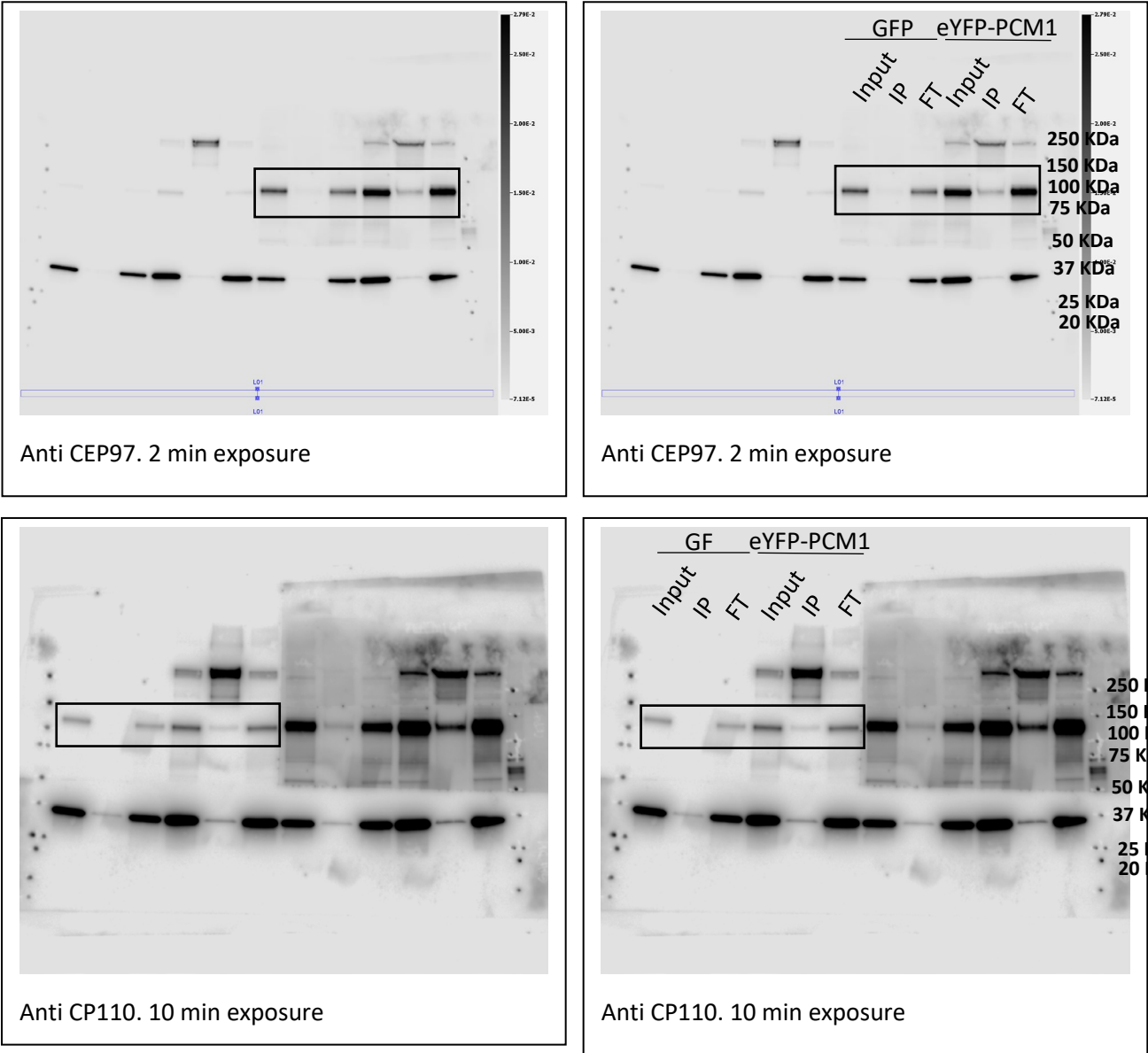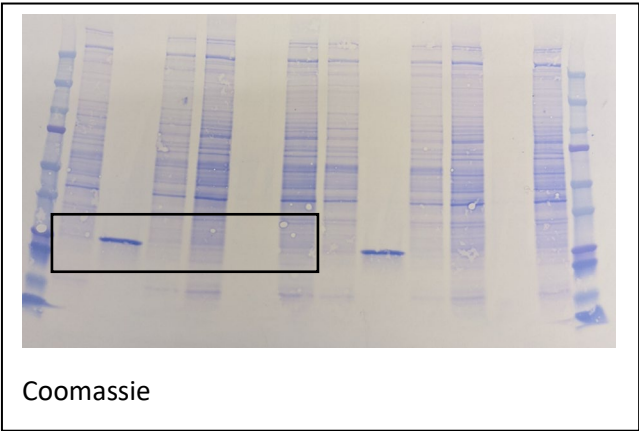

Figure 8- figure supplement 1E

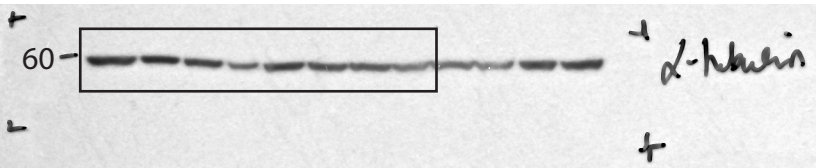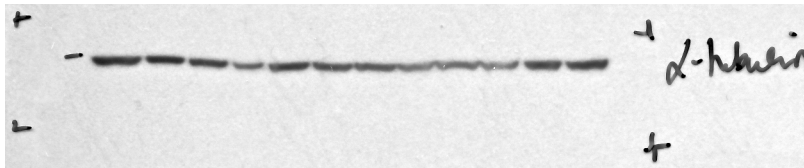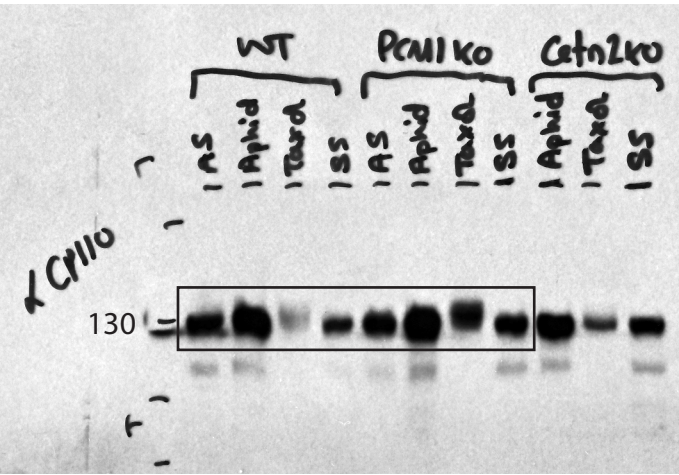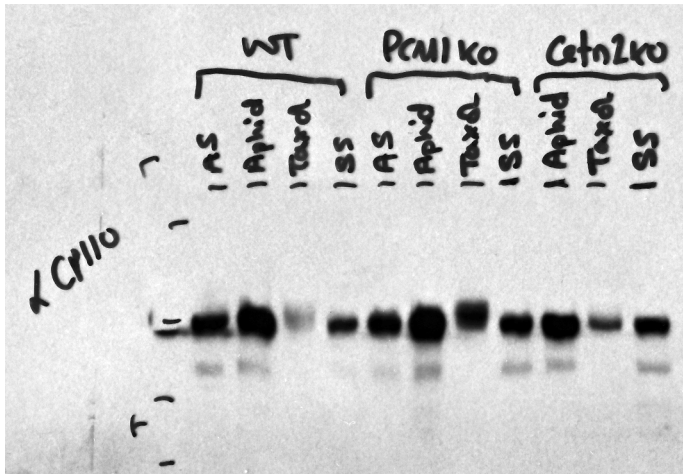

Supplement: Figure 8—source data 1. [file elife-79299-fig8-data1.pdf]
