# Supplementary material for: Centriolar satellites expedite mother centriole remodeling to promote ciliogenesis
Source: eLife. 2023 Feb 15;12:e79299. doi: 10.7554/eLife.79299 (PMC9998092; doi:10.7554/eLife.79299)
Supplement: Transparent reporting form [file elife-79299-transrepform1.docx]

***eLife’s* transparent reporting form**

We encourage authors to provide detailed information *within their submission* to facilitate the interpretation and replication of experiments. Authors can upload supporting documentation to indicate the use of appropriate reporting guidelines for health-related research (see [EQUATOR Network](http://www.equator-network.org/%20)), life science research (see the [BioSharing Information Resource](https://biosharing.org/)), or the [ARRIVE guidelines](http://www.plosbiology.org/article/info:doi/10.1371/journal.pbio.1000412) for reporting work involving animal research. Where applicable, authors should refer to any relevant reporting standards documents in this form.

If you have any questions, please consult our Journal Policies and/or contact us: [editorial@elifesciences.org](mailto:editorial@elifesciences.org).

**Sample-size estimation**

- You should state whether an appropriate sample size was computed when the study was being designed
- You should state the statistical method of sample size computation and any required assumptions
- If no explicit power analysis was used, you should describe how you decided what sample (replicate) size (number) to use

Please outline where this information can be found within the submission (e.g., sections or figure legends), or explain why this information doesn’t apply to your submission:

No power analysis was performed to compute the appropriate sample size for these studies. However, to support the robustness and reproducibility of our applications, we performed all experiments in at least 3 independent biological replicates.

Explicit details of the number of biological replicates and number of cells per sample imaged as well as proteomics analysis is mentioned in the respective figure legends.

**Replicates**

- You should report how often each experiment was performed
- You should include a definition of biological versus technical replication
- The data obtained should be provided and sufficient information should be provided to indicate the number of independent biological and/or technical replicates
- If you encountered any outliers, you should describe how these were handled
- Criteria for exclusion/inclusion of data should be clearly stated
- High-throughput sequence data should be uploaded before submission, with a private link for reviewers provided (these are available from both GEO and ArrayExpress)

Please outline where this information can be found within the submission (e.g., sections or figure legends), or explain why this information doesn’t apply to your submission:

Replicate information, including the number of experiments, biological and technical replicates, is provided in the figure legends. We use **biological replicate** to indicate an independent animal or animal-derived primary cell lines. **Technical replicate** is used to describe an independently treated sample in the same experiment. The number of data-points per condition is indicated in each sample, where each biological replicate is indicated as a separate symbol using SuperPlots. The complete proteome raw data is available on ProteomeXchange: PXD031920, as listed in Materials and Methods. All image analysis and measurement scripts are deposited on GitHub (<https://github.com/IGC-Advanced-Imaging-Resource/Hall2022_Paper>), as detailed in the Materials and Methods section.

**Statistical reporting**

- Statistical analysis methods should be described and justified
- Raw data should be presented in figures whenever informative to do so (typically when N per group is less than 10)
- For each experiment, you should identify the statistical tests used, exact values of N, definitions of center, methods of multiple test correction, and dispersion and precision measures (e.g., mean, median, SD, SEM, confidence intervals; and, for the major substantive results, a measure of effect size (e.g., Pearson's r, Cohen's d)
- Report exact p-values wherever possible alongside the summary statistics and 95% confidence intervals. These should be reported for all key questions and not only when the p-value is less than 0.05.

Please outline where this information can be found within the submission (e.g., sections or figure legends), or explain why this information doesn’t apply to your submission:

All statistical analyses used are described in the figure legends along with N numbers for biological replicates, n numbers for events and P values. Raw data for biological and experimental replicates has been plotted, where possible. Statistical methods used for analysis are mentioned in each figure as explained in Material and Methods.

(For large datasets, or papers with a very large number of statistical tests, you may upload a single table file with tests, Ns, etc., with reference to sections in the manuscript.)

**Group allocation**

- Indicate how samples were allocated into experimental groups (in the case of clinical studies, please specify allocation to treatment method); if randomization was used, please also state if restricted randomization was applied
- Indicate if masking was used during group allocation, data collection and/or data analysis

Please outline where this information can be found within the submission (e.g., sections or figure legends), or explain why this information doesn’t apply to your submission:

Experimental groups were assigned based on cell genotype. Care was taken to ensure images were taken at random by choosing fields of view based on DAPI, or a stain that was not subsequently being analyzed. In most cases, images were analyzed blinded to genotype for image analysis, and if possible, image analysis was automated to reduce bias.

**Additional data files (“source data”)**

- We encourage you to upload relevant additional data files, such as numerical data that are represented as a graph in a figure, or as a summary table
- Where provided, these should be in the most useful format, and they can be uploaded as “Source data” files linked to a main figure or table
- Include model definition files including the full list of parameters used
- Include code used for data analysis (e.g., R, MatLab)
- Avoid stating that data files are “available upon request”

Please indicate the figures or tables for which source data files have been provided:

Source Data for full labelled and unlabelled western blots is included see **Figure 1- source data 1, Figure 4 – source data 1 and Figure 8 – source data**, where all blots for a given figure and its supplements have been merged into a single PDF for ease . Proteomics data files are be uploaded ProteomeXchange (Identifier: PXD031920), with the accession number is available with the paper. All analysis tools have been made available on GitHub (<https://github.com/IGC-Advanced-Imaging-Resource/Hall2022_Paper>), as described in Materials and Methods.
